# Effects of dietary standardized ileal digestible crude protein levels on growth performance, meat quality, and Cecal microbiota of Arbor Acres broilers

**DOI:** 10.1016/j.psj.2026.107084

**Published:** 2026-05-06

**Authors:** Shurui Chen, Ruiping Liang, Min Fan, Lin Lu, Junyan Zhou

**Affiliations:** a College of Animal Science and Technology, Beijing University of Agriculture, Beijing, 102206, China; b Beijing Changping District Animal Disease Prevention and Control Center, Beijing, 102206, China

**Keywords:** Broiler, Standardized ileal digestible crude protein, Growth performance, Nutrient utilization

## Abstract

Crude protein (CP) is commonly used to characterize dietary nitrogen supply, but it does not account for ingredient-to-ingredient differences in protein digestibility and the importance of non-essential amino acids; thus, dietary standardized ileal digestible (SID) CP may provide a more precise basis for protein nutrition. Four independent experiments were conducted to investigate the effects of different SID CP levels on male Arbor Acres broilers at different growth stages (Exp. 1, 0–10 d; Exp. 2, 10–21 d; Exp. 3, 21–32 d; Exp. 4, 32–42 d). In each experiment, 175 one-d-old chicks were allotted to 5 graded SID CP levels with 7 replicates (5 broilers/replicate); SID CP level ranged from 17.5 to 21.5% during 0–21 d and 16.5–21.0% during 21–42 d, with other nutrients meeting NRC (1994) recommendations. Data were analyzed using general linear model (GLM) with each cage as the experimental unit, and dose responses were evaluated using orthogonal polynomial contrasts; the optimal SID CP level estimates were derived by regression when responses were significant. Increasing SID CP improved ADG and feed efficiency during 0–21 d with linear responses (*P* < 0.01). During 21–42 d, ADG responded quadratically to SID CP (*P* < 0.05), with optimal SID CP estimates of 19.00% (21–32 d; R² = 0.92) and 19.40% (32–42 d; R² = 0.99). Nutrient utilization (dry matter and crude protein) during 21–32 d exhibited significant linear and quadratic responses to SID CP (*P* < 0.05 to *P* < 0.01). Serum non-esterified fatty acids and pyruvic acid increased as SID CP increased (*P* < 0.01), whereas most other serum indices were unaffected. Carcass traits and key meat quality parameters were generally not compromised across SID CP levels. Cecal microbiota analysis revealed no significant differences in overall community diversity among treatment groups; however, SID CP altered the relative abundances of several specific genera and the composition of the microbial community. Specifically, as the SID CP level decreased from 21% to 18.75%, the abundance of *Ligilactobacillus* increased, and when the SID CP level was further reduced to 16.5%, the abundance of *Ruminococcus* decreased. Overall, SID CP levels of 19.0–19.4% optimize broiler of 21–42 d growth and nutrient utilization; higher SID CP offers no extra benefit but raises certain serum metabolites, whereas lower SID CP alters cecal microbiota without harming meat quality.

## Introduction

Efficient protein nutrition is essential for the performance of broilers and the utilization of nitrogen. However, crude protein (**CP**) serves only as a rough indicator of available nitrogen. This is because it fails to account for the significant differences in protein digestibility among various feed ingredients. Under current formulation practices, CP faces further challenges due to the increased use of supplementation with feed-grade amino acids in low-CP diets. Research has found that appropriately reducing dietary CP offers clear advantages, including lower feed costs, reduced nitrogen excretion, and improved environmental sustainability (Pomar and Remus, 2022; Kareem et al., 2025). However, it also presents other challenges. While the requirements for essential amino acids (**EAA**) may be met under these conditions, the total amount of digestible nitrogen may become limiting. This limitation can restrict the synthesis of non-essential amino acids (**NEAA**) and disrupt metabolic homeostasis (Santos-Sánchez et al., 2024). As a result, responses to low-CP programs are often inconsistent. This is largely because many alternative or by-product ingredients contain anti-nutritional factors. Compared to traditional ingredients like corn and soybean meal, their digestibility varies greatly, leading to an inaccurate estimation of available protein supply. Excessive reduction of dietary CP, particularly when not properly balanced with digestible nitrogen and amino acids, increases the risk of nitrogen deficiency. As a result, growth and feed efficiency can be negatively affected.

Dietary standardized ileal digestible (**SID**) CP provides an alternative framework to quantify usable protein supply. In the context of this study, SID CP refers to the protein fraction that is digested and absorbed before the terminal ileum. However, even when dietary CP levels are the same, the SID CP content can vary considerably. This variation is due to differences in ingredient composition and digestibility. Conceptually, SID CP incorporates information on both the digestibility of specific ingredients and the total nitrogen supply. It thus provides a more direct estimate of digestible nitrogen that supports both EAA utilization and NEAA synthesis. Therefore, incorporating SID CP into formulation may enhance the precision of low-CP diets reliant on feed-grade amino acids in meeting livestock and poultry requirements, by reducing the likelihood that total digestible nitrogen becomes limiting (Wu et al., 2013; Hou et al., 2015).

Broiler nutrient requirements need to be staged because priorities for physiological development, energy utilization, and nutrient partitioning shift decisively across the production cycle, driven by distinct changes in growth rate, body composition, and nutrient deposition patterns (An and Kong, 2025). Accordingly, the objective of this study was to determine the effects of SID CP levels on male broilers at different growth stages using feeding trials with graded SID CP levels, and to comprehensively assess the biological responses to SID CP supply.

## Materials and methods

This study followed the Chinese guidelines for experiments and animal welfare. All animal procedures were approved by the Animal Care Committee of Beijing University of Agriculture (Protocol No. BUA612506147, Beijing, China).

### Experimental design and feeding management

The experiment was conducted in March 2025 in the animal facility of Beijing University of Agriculture. Feeding and management were carried out according to the “Arbor Acres (AA) Broiler Performance Objectives” (Aviagen, 2022). The experiment was conducted using a cage rearing system. Prior to commencement, all equipment within the poultry house was thoroughly cleaned and disinfected. Vaccinations were administered at 7 and 21 d of age. From 0–7 d of age, the broilers were exposed to 23 hours of light daily. After 7 d of age, the photoperiod was gradually reduced, reaching 4 hours of darkness by d 28. During the first week (0–7 d of age), the house temperature was maintained between 32°C and 35°C. Subsequently, the temperature was reduced by 2°C–3°C each week. Relative humidity was maintained at 55%–60% throughout the trial. During the whole experiment, the broilers had free access to feed and water. This study was designed and consisted of four separate experiments, using growth performance as the main indicator, a regression-based approach was employed to assess the response of broilers to varying SID CP levels. In each experiment, a total of 175 male Arbor Acres broilers were selected, weighed, and randomly assigned to one of five graded SID CP levels, with seven replicates of 5 broilers each.

Experiment 1: Broilers aged 0–10 d were fed diets with five different SID CP levels (17.5%, 18.5%, 19.5%, 20.5%, 21.5%), all containing an apparent metabolizable energy (**AME**) of 2950 kcal/kg. Experiment 2: Broilers aged 10–21 d were fed diets with five different SID CP levels (17.5%, 18.5%, 19.5%, 20.5%, 21.5%), all containing an AME of 3030 kcal /kg. Experiment 3: Broilers aged 21–32 d were fed diets with five different SID CP levels (16.5%, 17.625%, 18.75%, 19.875%, 21%), all containing an AME of 3100 kcal /kg. Experiment 4: Broilers aged 32–42 d were fed diets with five different SID CP levels (16.5%, 17.625%, 18.75%, 19.875%, 21%), all containing an AME of 3180 kcal /kg.

The SID values of protein for all feed ingredients used in the diet formulation were based on the following references: the National Research Council standards (National Research Council, 1994), the Chinese Feed Composition and Nutritional Value Table (35th edition) (Institute of Animal Sciences, Chinese Academy of Agricultural Sciences, Beijing et al., 2024), and the CVB Veevoedertabel 2023 (Centraal Veevoeder Bureau, 2023). Specific data are provided in Table 1. During the growing phase, adequate intake of EAA is critical for the growth performance and health of broilers. In this study, the basal SID EAA levels in each dietary treatment were first calculated based on the SID EAA contents of the feed ingredients. Subsequently, feed-grade amino acids were supplemented to ensure that digestible EAA levels were consistent across all treatment groups, thereby conforming to the nutritional recommendations of NRC (1994). Furthermore, studies have shown that glycine is the most critical limiting NEAA in low-protein diets for broilers (Awad et al., 2015). When dietary crude protein is too low, insufficient glycine supply can significantly impair growth performance (Liu et al., 2024). Therefore, glycine levels were balanced across all treatment groups to ensure that each experimental diet met the broilers’ glycine requirements. The composition and estimated nutrient contents of all experimental diets are shown in Table 2, Table 3, Table 4, Table 5. All experimental diets were formulated using the same batch of ingredients. Chromium oxide (Cr₂O₃) was added at 0.3% to the diet of broilers aged 21–32 d as an inert marker for determining the apparent ileal digestibility of amino acids. On the final day of each of the four trials, average daily gain (**ADG**), average daily feed intake (**ADFI**), and gain-to-feed ratio (**G/F**) were recorded and calculated. Three treatment groups were selected for subsequent slaughter: the high SID CP (**HP**) group with an SID CP level of 21%, the medium SID CP (**MP**) group with an SID CP level of 18.75%, and the low SID CP (**LP**) group with an SID CP level of 16.5%.

**Table 1:** Nutritional value of feed ingredients.

Feed ingredients	Corn	wheat	Soybean meal	Broken rice	Corn gluten meal	Field pea	Flour	Soybean oil
AME^1^, kcal/kg	3090	2870	2490	3350	3440	2640	3120	8800
CP^2^, %	8.00	11.00	43.90	7.80	60.40	20.10	14.10	-
SID^3^ CP, %	7.30	9.51	37.85	7.10	50.46	16.87	12.13	-
SID^3^ Lys, %	0.21	0.25	2.40	0.32	0.81	1.26	0.38	-
SID^3^ Met, %	0.16	0.16	0.55	0.15	1.29	0.17	0.20	-
Ca, %	0.02	0.10	0.27	0.06	0.03	0.09	0.04	-
SID^3^ P, %	0.12	0.20	0.27	0.15	0.32	0.21	0.34	-
SID^3^ Arg, %	0.35	0.43	2.94	0.57	1.66	1.59	0.65	-
SID^3^ Trp, %	0.04	0.12	0.51	0.08	0.22	0.14	0.17	-
SID^3^ SAA^4^, %	0.31	0.37	1.04	0.29	2.10	0.39	0.44	-
SID^3^ Thr, %	0.23	0.26	1.41	0.27	1.62	0.60	0.34	-
SID^3^ Ile, %	0.26	0.33	1.76	0.28	2.11	0.68	0.47	-
SID^3^ Cys, %	0.15	0.21	0.49	0.14	0.81	0.22	0.24	-
SID^3^ Val, %	0.34	0.41	1.74	0.40	2.39	0.76	0.57	-
SID^3^ Leu, %	0.91	0.65	2.92	0.53	8.93	1.19	0.84	-
SID^3^ Tyr, %	0.26	0.26	1.42	0.25	0.00	0.56	0.37	-
SID^3^ Phe, %	0.35	0.45	1.99	0.33	3.35	0.78	0.57	-
SID^3^ His, %	0.21	0.21	1.03	0.19	1.04	0.43	0.29	-
SID^3^ Gly, %	0.27	0.37	1.59	0.37	1.21	0.74	0.50	-

^5^CP contents of feed ingredients were obtained from the Chinese Feed Composition and Nutritional Value Table (35th edition, 2024). SID values of protein and amino acids were obtained from CVB Veevoedertabel (2023).

1 AME, Apparent metabolism energy.

2 CP, crude protein.

3 SID, standardized ileal digestible.

4 SAA, methionine + cysteine.

**Table 2:** Ingredient composition of the diets of 0**–**10 d broilers (as-fed basis).

Items	SID CP content, %				
	21.50	20.50	19.50	18.50	17.50
Ingredients, %					
Corn	32.94	36.95	40.94	45.34	50.19
Flour	5.00	5.00	5.00	5.00	5.00
Wheat	2.00	2.00	2.00	2.00	2.00
Pea	2.00	2.00	2.00	2.00	2.00
Corn gluten meal	8.00	8.00	8.00	8.00	8.00
Broken rice	10.00	10.00	10.00	10.00	10.00
Soybean meal	32.33	27.90	23.48	18.91	13.85
Soybean oil	2.34	1.94	1.54	1.03	0.40
Salt	0.35	0.35	0.35	0.35	0.35
Limestone	1.35	1.35	1.35	1.35	1.35
Dicalcium phosphate	1.56	1.61	1.65	1.69	1.74
L-Lysine·HCl	0.52	0.64	0.77	0.89	1.04
DL-Methionine	0.43	0.47	0.50	0.54	0.57
L-Threonine	0.21	0.27	0.32	0.38	0.44
L-Valine	0.11	0.17	0.24	0.31	0.38
L-Isoleucine	0.02	0.09	0.16	0.23	0.30
L-Arginine	0.12	0.24	0.36	0.48	0.62
L-Glycine	0.02	0.08	0.14	0.21	0.28
L-Tryptophan	0.03	0.05	0.08	0.10	0.12
L-Phenylalanine	-	-	-	-	0.14
L-Histidine	-	-	-	0.02	0.06
Antifungal agent^1^	0.04	0.04	0.04	0.04	0.04
Choline chloride	0.10	0.10	0.10	0.10	0.10
Complex enzyme^2^	0.03	0.03	0.03	0.03	0.03
Potassium magnesium sulfate	-	0.22	0.45	0.50	0.50
Premix^3^	0.50	0.50	0.50	0.50	0.50
Total	100	100	100	100	100
Calculated values					
AME^4^, kcal/kg	2950	2950	2950	2950	2950
Crude protein, %	24.88	23.68	22.47	21.26	20.04
Ca, %	0.94	0.94	0.94	0.94	0.94
Digestible phosphorus, %	0.45	0.45	0.45	0.45	0.45
SID Lys, %	1.40	1.40	1.40	1.40	1.40
SID SAA, %	1.10	1.10	1.10	1.10	1.10
SID Arg, %	1.45	1.45	1.45	1.45	1.45
SID Trp, %	0.25	0.25	0.25	0.25	0.25
SID Thr, %	0.93	0.93	0.93	0.93	0.93
SID Ile, %	0.92	0.92	0.92	0.92	0.92
SID Val, %	1.06	1.06	1.06	1.06	1.06
SID Gly, %	0.80	0.80	0.80	0.80	0.80
SID Phe+Tyr, %	1.72	1.59	1.46	1.33	1.33
SID Leu, %	2.09	1.99	1.90	1.81	1.70
SID His, %	0.53	0.50	0.46	0.44	0.44
Analyzed nutrient levels, %					
Dry matter	91.00	90.65	90.93	90.16	90.23
Crude protein	25.14	23.96	22.54	21.66	20.47
Crude ash	6.52	6.38	6.43	6.19	6.04

1 Antifungal agent: propionic acid, 40.0%; Sodium propionate, 10.0%; carrier: silicon dioxide.

2 Complex enzyme: Enzymes: cellulase, 5000 U/g; xylanase, 24000 U/g; neutral protease, 3000 U/g; acid protease, 3000 U/g. Carrier: rice husk powder, medical stone.

3 Premix provided the following per kg of complete diet for growing broilers: vitamin A, 1500 KIU; vitamin D3, 500 KIU; vitamin E, 5000 IU; vitamin K3, 400 mg; vitamin B1, 300 mg; vitamin B2, 1200 mg; vitamin B6, 600 mg; vitamin B12, 2.4 mg; nicotinamide, 8000 mg; pantothenic acid, 2000 mg; folic acid, 160 mg; biotin, 20 mg; Mn, 14 g; Fe, 16 g; Zn, 10 g; Cu, 1.5 g; I, 100 mg; Se, 30 mg.

4 AME, Apparent metabolism energy.

**Table 3:** Ingredient composition of the diets of 10**–**21 d broilers (as-fed basis).

Items	SID CP content, %				
	21.50	20.50	19.50	18.50	17.50
Ingredients, %					
Corn	28.33	32.17	36.17	40.51	45.07
Flour	5.00	5.00	5.00	5.00	5.00
Wheat	2.00	2.00	2.00	2.00	2.00
Pea	2.00	2.00	2.00	2.00	2.00
Corn gluten meal	8.00	8.00	8.00	8.00	8.00
Broken rice	13.00	13.00	13.00	13.00	13.00
Soybean meal	33.68	29.52	25.12	20.62	15.94
Soybean oil	3.59	3.23	2.84	2.34	1.78
Salt	0.35	0.35	0.35	0.35	0.35
Limestone	1.35	1.35	1.35	1.35	1.35
Dicalcium phosphate	1.12	1.16	1.21	1.25	1.29
L-Lysine·HCl	0.36	0.48	0.60	0.73	0.86
DL-Methionine	0.35	0.39	0.42	0.45	0.49
L-Threonine	0.16	0.21	0.26	0.32	0.37
L-Valine	0.03	0.09	0.16	0.22	0.29
L-Isoleucine	-	-	0.06	0.13	0.20
L-Arginine	-	0.10	0.21	0.33	0.46
L-Glycine	-	0.04	0.10	0.16	0.22
L-Tryptophan	0.01	0.03	0.05	0.07	0.10
L-Phenylalanine	-	-	-	-	0.03
L-Histidine	-	-	-	-	0.03
Antifungal agent^1^	0.04	0.04	0.04	0.04	0.04
Choline chloride	0.10	0.10	0.10	0.10	0.10
Complex enzyme^2^	0.03	0.03	0.03	0.03	0.03
Potassium magnesium sulfate	-	0.21	0.43	0.50	0.50
Premix^3^	0.50	0.50	0.50	0.50	0.50
Total	100	100	100	100	100
Calculated values					
AME^4^, kcal/kg	3030	3030	3030	3030	3030
Crude protein, %	24.91	23.71	22.51	21.31	20.09
Ca, %	0.85	0.84	0.84	0.84	0.84
Digestible phosphorus, %	0.38	0.38	0.38	0.38	0.38
SID Lys, %	1.30	1.30	1.30	1.30	1.30
SID SAA, %	1.03	1.03	1.03	1.03	1.03
SID Arg, %	1.37	1.35	1.35	1.35	1.35
SID Trp, %	0.23	0.23	0.23	0.23	0.23
SID Thr, %	0.88	0.88	0.88	0.88	0.88
SID Ile, %	0.91	0.85	0.84	0.84	0.84
SID Val, %	1.00	1.00	1.00	1.00	1.00
SID Gly, %	0.80	0.78	0.78	0.78	0.78
SID Phe+Tyr, %	1.75	1.63	1.50	1.38	1.27
SID Leu, %	2.10	2.01	1.92	1.83	1.73
SID His, %	0.54	0.51	0.47	0.44	0.42
Analyzed nutrient levels, %					
Dry matter	90.68	90.41	88.44	89.82	90.57
Crude protein	25.12	24.01	22.91	21.77	20.33
Crude ash	6.36	6.30	5.83	5.94	6.19

1 Antifungal agent: propionic acid, 40.0%; Sodium propionate, 10.0%; carrier: silicon dioxide.

2 Complex enzyme: Enzymes: cellulase, 5000 U/g; xylanase, 24000 U/g; neutral protease, 3000 U/g; acid protease, 3000 U/g. Carrier: rice husk powder, medical stone.

3 Premix provided the following per kg of complete diet for growing broilers: vitamin A, 1500 KIU; vitamin D3, 500 KIU; vitamin E, 5000 IU; vitamin K3, 400 mg; vitamin B1, 300 mg; vitamin B2, 1200 mg; vitamin B6, 600 mg; vitamin B12, 2.4 mg; nicotinamide, 8000 mg; pantothenic acid, 2000 mg; folic acid, 160 mg; biotin, 20 mg; Mn, 14 g; Fe, 16 g; Zn, 10 g; Cu, 1.5 g; I, 100 mg; Se, 30 mg.

4 AME, Apparent metabolism energy.

**Table 4:** Ingredient composition of the diets of 21**–**32 d broilers (as-fed basis).

Items	SID CP content, %								
	21.00		19.875		18.75		17.625		16.50
Ingredients, %									
Corn	24.95	29.02		33.44		38.37		43.45	
Flour	5.00	5.00		5.00		5.00		5.00	
Wheat	2.00	2.00		2.00		2.00		2.00	
Pea	2.00	2.00		2.00		2.00		2.00	
Corn gluten meal	8.00	8.00		8.00		8.00		8.00	
Broken rice	17.00	17.00		17.00		17.00		17.00	
Soybean meal	32.84	28.61		23.76		18.68		13.51	
Soybean oil	4.43	4.09		3.65		3.08		2.47	
Salt	0.35	0.35		0.35		0.35		0.35	
Limestone	1.35	1.35		1.35		1.35		1.35	
Dicalcium phosphate	0.82	0.86		0.91		0.96		1.00	
L-Lysine·HCl	0.24	0.36		0.50		0.64		0.78	
DL-Methionine	0.28	0.31		0.35		0.39		0.43	
L-Threonine	0.07	0.12		0.18		0.25		0.31	
L-Valine	-	0.01		0.09		0.16		0.24	
L-Isoleucine	-	-		0.03		0.11		0.19	
L-Arginine	-	0.02		0.15		0.28		0.42	
L-Glycine	-	0.01		0.08		0.15		0.22	
L-Tryptophan	-	0.01		0.03		0.06		0.08	
L-Phenylalanine	-	-		-		-		0.02	
L-Histidine	-	-		-		-		0.01	
Antifungal agent^1^	0.04	0.04		0.04		0.04		0.04	
Choline chloride	0.10	0.10		0.10		0.10		0.10	
Complex enzyme^2^	0.03	0.03		0.03		0.03		0.03	
Potassium magnesium sulfate	-	0.21		0.46		0.50		0.50	
Premix^3^	0.50	0.50		0.50		0.50		0.50	
Total	100	100		100		100		100	
Calculated values									
AME^4^, kcal/kg	3100	3100		3100		3100		3100	
Crude protein, %	24.33	23.01		21.66		20.30		18.94	
Ca, %	0.77	0.77		0.77		0.77		0.77	
Digestible phosphorus, %	0.33	0.33		0.33		0.33		0.33	
SID SAA, %	0.95	0.95		0.95		0.95		0.95	
SID Lys, %	1.20	1.20		1.20		1.20		1.20	
SID Arg, %	1.36	1.26		1.26		1.26		1.26	
SID Trp, %	0.22	0.21		0.21		0.21		0.21	
SID Thr, %	0.80	0.80		0.80		0.80		0.80	
SID Ile, %	0.90	0.84		0.79		0.79		0.79	
SID Val, %	0.97	0.92		0.92		0.92		0.92	
SID Gly, %	0.80	0.75		0.75		0.75		0.75	
SID Phe+Tyr, %	1.72	1.61		1.47		1.32		1.20	
SID Leu, %	2.07	1.98		1.88		1.77		1.67	
SID His, %	0.53	0.50		0.46		0.42		0.38	
Analyzed nutrient levels, %									
Dry matter	90.74	90.58		90.65		89.60		89.99	
Crude protein	24.64	23.38		21.78		20.67		18.88	
Crude ash	6.36	5.85		6.09		5.72		5.27	

1 Antifungal agent: propionic acid, 40.0%; Sodium propionate, 10.0%; carrier: silicon dioxide.

2 Complex enzyme: Enzymes: cellulase, 5000 U/g; xylanase, 24000 U/g; neutral protease, 3000 U/g; acid protease, 3000 U/g. Carrier: rice husk powder, medical stone.

3 Premix provided the following per kg of complete diet for growing broilers: vitamin A, 1500 KIU; vitamin D3, 500KIU; vitamin E, 5000 IU; vitamin K3, 400 mg; vitamin B1, 300 mg; vitamin B2, 1200 mg; vitamin B6, 600 mg; vitamin B12, 2.4 mg; nicotinamide, 8000 mg; pantothenic acid, 2000 mg; folic acid, 160 mg; biotin, 20 mg; Mn, 14 g; Fe, 16 g; Zn, 10 g; Cu, 1.5 g; I, 100 mg; Se, 30 mg.

4 AME, Apparent metabolism energy.

**Table 5:** Ingredient composition of the diets of 32**–**42 d broilers (as-fed basis).

Items	SID CP content, %				
	21.00	19.875	18.75	17.625	16.50
Ingredients, %					
Corn	20.22	24.24	28.22	32.92	37.93
Flour	5.00	5.00	5.00	5.00	5.00
Wheat	2.00	2.00	2.00	2.00	2.00
Pea	2.00	2.00	2.00	2.00	2.00
Corn gluten meal	8.00	8.00	8.00	8.00	8.00
Broken rice	20.00	20.00	20.00	20.00	20.00
Soybean meal	33.76	29.64	25.53	20.72	15.64
Soybean oil	5.73	5.40	5.08	4.57	3.97
Salt	0.35	0.35	0.35	0.35	0.35
Limestone	1.35	1.35	1.35	1.35	1.35
Dicalcium phosphate	0.63	0.67	0.71	0.75	0.80
L-Lysine·HCl	0.09	0.20	0.32	0.45	0.59
DL-Methionine	0.20	0.23	0.26	0.29	0.33
L-Threonine	-	0.05	0.10	0.16	0.22
L-Valine	-	-	-	0.06	0.13
L-Isoleucine	-	-	-	0.01	0.09
L-Arginine	-	-	-	0.12	0.25
L-Glycine	-	-	-	0.06	0.13
L-Tryptophan	-	-	-	0.02	0.05
Antifungal agent^1^	0.04	0.04	0.04	0.04	0.04
Choline chloride	0.10	0.10	0.10	0.10	0.10
Complex enzyme^2^	0.03	0.03	0.03	0.03	0.03
Potassium magnesium sulfate	-	0.20	0.41	0.50	0.50
Premix^3^	0.50	0.50	0.50	0.50	0.50
Calculated values					
AME^4^, kcal/kg	3180	3180	3180	3180	3180
Crude protein, %	24.33	23.02	21.70	20.35	19.00
Ca, %	0.73	0.73	0.73	0.73	0.73
Digestible phosphorus, %	0.30	0.30	0.30	0.30	0.30
SID Lys, %	1.10	1.10	1.10	1.10	1.10
SID SAA, %	0.87	0.87	0.87	0.87	0.87
SID Arg, %	1.38	1.28	1.17	1.16	1.16
SID Trp, %	0.23	0.21	0.19	0.19	0.19
SID Thr, %	0.74	0.74	0.74	0.74	0.74
SID Ile, %	0.91	0.85	0.79	0.73	0.73
SID Val, %	0.98	0.92	0.86	0.85	0.85
SID Gly, %	0.81	0.75	0.70	0.69	0.69
SID Phe+Tyr, %	1.75	1.63	1.51	1.38	1.23
SID Leu, %	2.07	1.98	1.90	1.80	1.70
SID His, %	0.54	0.51	0.47	0.43	0.39
Analyzed nutrient levels, %					
Dry matter	90.44	89.64	90.91	90.12	90.45
Crude protein	24.52	23.20	22.03	20.14	19.10
Crude ash	4.14	5.38	5.22	5.13	4.96

1 Antifungal agent: propionic acid, 40.0%; Sodium propionate, 10.0%; carrier: silicon dioxide.

2 Complex enzyme: Enzymes: cellulase, 5000 U/g; xylanase, 24000 U/g; neutral protease, 3000 U/g; acid protease, 3000 U/g. Carrier: rice husk powder, medical stone.

3 Premix provided the following per kg of complete diet for growing broilers: vitamin A, 1500 KIU; vitamin D3, 500 KIU; vitamin E, 5000 IU; vitamin K3, 400 mg; vitamin B1, 300 mg; vitamin B2, 1200 mg; vitamin B6, 600 mg; vitamin B12, 2.4 mg; nicotinamide, 8000 mg; pantothenic acid, 2000 mg; folic acid, 160 mg; biotin, 20 mg; Mn, 14 g; Fe, 16 g; Zn, 10 g; Cu, 1.5 g; I, 100 mg; Se, 30 mg.

4 AME, Apparent metabolism energy.

### Sample collection

Prior to the experiment, feed from each experimental group (500 g) was collected following standard procedures (GB/T 14699.1–2005) and stored at 4°C. Excreta samples were collected from broilers aged 21–32 d during the final three days of Experiment 3. Collection was performed using metabolism cages, and samples were stored at −20°C until subsequent analysis of apparent nutrient metabolic rates. At 43 d of age, after a 12-hour fasting period, individual birds were weighed. One broiler per replicate was selected based on average body weight. Blood was collected from the wing vein, and the broiler was subsequently euthanized by exsanguination via the jugular vein. The collected blood samples were allowed to stand at room temperature for 30 minutes, then centrifuged at 4°C and 3000 × g for 10 minutes. The serum was separated and stored at −20°C for subsequent analysis. From each of the three experimental slaughter groups (HP, MP, and LP), 6 broilers were selected based on average body weight and subsequently euthanized by exsanguination via the jugular vein. The left pectoral muscle was excised and stored at 4°C for carcass trait measurement. Under aseptic conditions, approximately 1–2 grams of digesta from the middle cecum was collected into a sterile cryotube. Immediately after collection, samples were flash-frozen in liquid nitrogen and subsequently transferred to a − 80°C ultra-low temperature freezer for long-term storage until subsequent analysis of cecal microbial diversity.

### Growth performance and slaughter characteristics

Throughout the experiment, the health status and mortality of individual birds were observed daily, and the number of deaths was recorded. On the final day of each of the four trials, broilers were weighed individually, and feed consumption per pen was recorded to calculate ADG, ADFI, and G/F. One additional broiler per replicate was selected based on the average body weight for carcass evaluation. According to the Chinese agricultural industry standard “Performance terminology and measurements for poultry” (NY/T 823–2020), the slaughter performance of broilers was evaluated by measuring the dressing percentage and eviscerated yield rate.

### Nutrient digestibility

Feed samples from animals aged 21–32 d were collected for the determination of nutritional levels in the basal diet. Excreta samples were collected using the metabolic cage method during the last 3 d of the 21–32 d age period for the determination of nutritional levels in excreta. The dry matter (**DM**) content was measured using the method GB procedure (GB/T 6435–2014); the CP content was determined using a standardized methodology (GB/T 6432–2018); the crude ash content was determined according to the GB method (GB/T 6438–2007); and the hydrochloric acid-insoluble ash content was determined strictly following the standardized testing procedure (GB/T 23742–2009).

### Carcass Traits and Meat Quality

Drip loss (NY/T 1333–2007): Three pieces of meat, each measuring 1 cm × 1 cm × 2 cm, were cut from every sample. After recording the initial weight (*m_1_*), the samples were hung in a sealed drip loss container and stored at 4°C for 24 h. They were then taken out and weighed again to obtain the final weight (*m_2_*).

Cooking loss (NY/T 2793–2015): A whole meat sample of approximately 30 g was accurately weighed using a balance and recorded as *m_3_*. The sample was then placed in a self-sealing bag and labeled properly, followed by immersion in a constant-temperature water bath set at 80°C for 45 min. After removal, the meat sample was allowed to cool naturally to room temperature, and its surface moisture was immediately blotted dry with filter paper before re-weighing. The weight measured at this point was recorded as *m_4_*.

Shear force (NY/T 1180–2006): A meat sample was taken and cooked in water at 75°C for 30 min before being removed. The sample was then cut into strips with dimensions of 1 cm × 1 cm × 3 cm, with the muscle fiber direction kept parallel to the length direction of the strips. Three strips were measured per sample, and the average value was calculated.

### Serum biochemical indices

On d 43, the individual birds that had been fasted for 12 hours were weighed, and one chicken was selected from each replicate based on the average body weight. The serum was separated by centrifugation at 3000 × g for 10 minutes under 4°C conditions, and then stored at −20°C. Routine serum biochemical indices were determined using commercial kits (Nanjing Jiancheng Bioengineering Institute, China) and an automatic biochemistry analyzer (BS-420, Mindray, China). The serum biochemical indices included glucose (**GLU**), total protein (**TP**), albumin (**ALB**), urea nitrogen (**UN**), total cholesterol (**TC**), triglycerides (**TG**), pyruvic acid (**Pyr**), total amino acids (**TAAs**), and free fatty acids (**FFA**).

### DNA extraction and microbial 16S rRNA gene sequencing analysis

Total microbial genomic DNA was extracted from cecal chyme samples using the E.Z.N.A.® soil DNA Kit (Omega Bio-tek, Norcross, GA, U.S.) following the manufacturer's instructions. The quality and concentration of DNA were examined using 1.0% agarose gel electrophoresis and a NanoDrop2000 spectrophotometer (Thermo Scientific, USA). The samples were then stored at −80°C until needed. To study the bacteria, we amplified the V3-V4 region of their 16S rRNA gene. We used the primers 338F and 806R and ran the PCR reaction in a T100 Thermal Cycler (BIO-RAD, USA) (Liu et al., 2016). The PCR product was extracted from 2% agarose gel and purified with the PCR Clean-Up Kit (YuHua, Shanghai, China), following the kit's instructions. Its concentration was then measured using a Qubit 4.0 instrument (Thermo Fisher Scientific, USA). The sequencing work was done by Majorbio Bio-Pharm Technology Co., Ltd. (Shanghai, China) using the Illumina NovaSeq PE250 platform. Subsequently, all bioinformatics analyses were performed on the Majorbio Cloud Platform (https://cloud.majorbio.com/page/tools/). Based on the OTUs information, alpha diversity indices, including observed OTUs, Chao index, Sobs index, Shannon index, and Simpson index, were calculated using Mothur v1.30.1 (Schloss et al., 2009). The similarity among microbial communities in different samples was determined by Principal Component Analysis (**PCA**), and the significance of the changes in community structure was assessed by ANOSIM test (*P* < 0.05). The Wilcoxon rank-sum test was used to analyze significant differences in species between different samples.

### Data computation and analysis

▒

G/F= (Final body weight − Initial body weight) / Feed intake

Dressing percentage (%) = Dressed weight / Live weight × 100%

Eviscerated yield rate (%) = Eviscerated weight / Live weight × 100%

Apparent digestibility (%) = [1 − (Indicator content in feed × Nutrient content in excreta) / (Indicator content in excreta × Nutrient content in feed)] × 100%

Drip loss (%) = [(*m_1_* – *m_2_*) / *m_1_*] × 100%

Cooking loss (%) = [(*m_3_* – *m_4_*) / *m_3_*] × 100%

All data were analyzed using SAS 9.4 (SAS Institute Inc., Cary, North Carolina, USA). Prior to analysis, the Shapiro-Wilk test (univariate procedure) was used to verify the normality of the distribution, and the Levene’s test was performed to assess the homogeneity of variances. No significant violations of normality or homoscedasticity were detected (*P* > 0.05). The statistical model was defined as follows:

$$Y_{\text{ij}}=\mu +T_{i}+e_{\text{ij}}$$

In the model, Yij represents the dependent variable (ADG, ADFI, G/F, or plasma biochemical indices); *μ* is the overall mean; *T*_i_ denotes the fixed effect of dietary SID CP level (*i* = 5); and *e_ij_* is the random error term. Each treatment consisted of *j* = 6 replicates, which served as the experimental unit.

We used the general linear model (**GLM**) to analyze the data for ADG, ADFI, G/F, and blood biochemical markers. Mean separation was performed exclusively using orthogonal polynomial contrasts to check whether the response to different SID CP levels followed a straight-line (linear) pattern or a curved (quadratic) pattern. These contrasts were set up using coefficients based on the spacing between the treatment levels. A *P*-value ≤0.05 was considered statistically significant, with smaller *P*-values indicating stronger evidence against the null hypothesis.

Pooled standard error of the mean (**SEM**) was calculated from the mean square error (**MSE**) of the GLM as SEM=$\surd (\frac{MSE}{n})$, where n = 6. The pooled SEM is reported in tables.

The SID CP levels required to meet the nutritional needs of broilers were estimated using broken-line regression model (**BLM**) and quadratic regression model (**QRM**), with biological interpretations and selection criteria referenced to Baker (Baker, 1986). These regression models were used only for requirement estimation, not as a mean separation procedure:

BLM: Describes a linear response of performance to SID CP until a plateau, representing the average requirement for most broilers (minimum SID CP to achieve plateau growth). The model was structured as:

Y = a + b(X – X_0_) (if X ≤ X_0_); Y = a (if X > X_0_)

where X_0_ = optimal SID CP requirement (break point), a = plateau value of Y, b = linear coefficient before plateau, and X = dietary SID CP level.

QRM: Captures curvilinear responses, estimating SID CP for the entire population to reach maximum performance. To avoid overestimation, 95% of the maximum response (95% MR) was used for practical requirement conversion. The model was:

Y = c + dX + eX^2^

where c = intercept, d = linear coefficient, e = quadratic coefficient. Maximum Y (ADG: max gain; G/F: max ratio) was calculated as X_max_ = -d/(2e), and 95% MR requirement as X_95%MR_ = Xmax - 0.1 × (X_max_ - X_min_) (where X_min_ = lowest SID CP with significant response).

## Result

### Growth performance

**Fig. 1:**
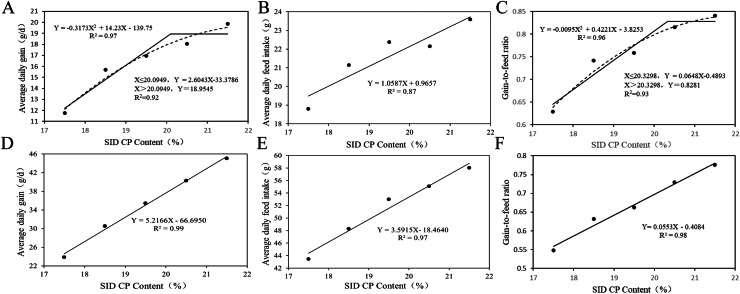
Analysis of optimal SID CP level of 0–21 d Broilers. Broken-line regression and quadratic models were fitted to describe the changes in ADG (A) and G/F (C) of broilers aged 0–10 d as a function of SID CP content. Linear regression analysis was performed for ADFI (B) of broilers aged 0–10 d, and for ADG (D), ADFI (E), and G/F (F) of broilers aged 10–21 d, to evaluate the effects of SID CP levels. For ADFI of broilers aged 0–10 d, as well as for ADG, ADFI, and G/F of broilers aged 10–21 d, no significant quadratic responses were observed (*P* > 0.05); thus, precluding the application of broken-line and quadratic regression model analyses.

As shown in Table 6 and Fig. 1, for broilers aged 0–10 d, ADG (Fig. 1‑A) and G/F (Fig. 1‑C) showed linear (*P*_linear_ <0.01) and quadratic (*P*_quadratic_ <0.05) responses to increasing SID CP levels. The SID CP level at the plateau breakpoint for ADG was 20.09% (R² = 0.92), and the SID CP level corresponding to the optimal ADG was 22.42% (R² = 0.97). The SID CP level at the plateau breakpoint for G/F was 20.33% (R² = 0.93), and the SID CP level corresponding to the optimal G/F was 22.22% (R² = 0.96). It should be noted that 22.42% and 22.22% value are extrapolated from the quadratic model beyond the highest dietary SID CP level tested in this experiment. Increasing SID CP levels resulted in linear increases (*P*_linear_ <0.01) in ADFI of broilers aged 0–10 d (Fig. 1‑B), as well as in ADG (Fig. 1-D), ADFI (Fig. 1-E), and G/F (Fig. 1-F) of broilers aged 10–21 d.

**Table 6:** The impact of SID CP content on the growth performance of 0**–**42 d broilers.

Items		SID CP levels					SEM^1^	P-value	
		Low	Sub Low	Middle	Sub High	High		Linear	Quadratic
1**–**10 d	SID CP levels	21.50	20.50	19.50	18.50	17.50			
	ADG, g	19.86	18.05	16.96	15.69	11.75	0.53	<0.01	0.02
	ADFI, g	23.59	22.15	22.37	21.14	18.80	0.36	<0.01	0.10
	G/F	0.84	0.82	0.76	0.74	0.63	0.15	<0.01	0.04
11**–**21 d	SID CP levels	21.50	20.50	19.50	18.50	17.50			
	ADG, g	45.05	40.29	35.42	30.53	23.85	1.65	<0.01	0.65
	ADFI, g	58.03	55.07	53.00	48.30	43.46	1.44	<0.01	0.52
	G/F	0.78	0.73	0.66	0.63	0.55	0.02	<0.01	0.59
22**–**31 d	SID CP levels	21.00	19.875	18.75	17.625	16.50			
	ADG, g	60.01	67.90	65.72	64.54	57.03	1.58	0.35	0.03
	ADFI, g	94.81	106.15	106.98	102.65	102.41	2.71	0.28	0.12
	G/F	0.63	0.63	0.62	0.59	0.55	0.01	<0.01	0.01
32**–**42 d	SID CP levels	21.00	19.875	18.75	17.625	16.50			
	ADG, g	75.74	80.84	79.69	74.79	64.52	2.09	0.04	0.04
	ADFI, g	129.38	130.46	141.67	144.37	139.24	2.40	0.05	0.27
	G/F	0.58	0.58	0.55	0.54	0.51	0.01	<0.01	0.56

1 Standard error of the mean, n = 6.

**Fig. 2:**
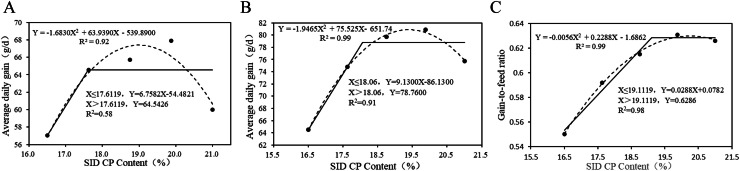
Analysis of optimal SID CP level of 21–42 d Broilers. Broken-line regression and quadratic models were fitted to describe the changes in ADG (A) and G/F (B) of broilers aged 21–32 d, as well as the G/F (C) of broilers aged 32–42 d, as a function of SID CP content. For ADFI of broilers aged 21–32 d, as well as for ADG and ADFI of broilers aged 32–42 d, no significant quadratic responses were observed (*P* > 0.05); thus, precluding the application of broken-line and quadratic regression model analyses.

The growth performance results for broilers aged 21–42 d are presented in Table 6 and Fig. 2. For broilers aged 21–32 d, ADG (Fig. 2‑A) showed a quadratic response (*P*_quadratic_ <0.05) to increasing SID CP levels, with the SID CP level corresponding to the optimal ADG being 19.00% (R² = 0.92). The G/F (Fig. 2‑B) of broilers aged 21–32 d showed linear (*P*_linear_ <0.01) and quadratic (*P*_quadratic_ = 0.01) responses to increasing SID CP levels. The SID CP level at the plateau breakpoint for G/F was 19.11% (R² = 0.98), and the SID CP level corresponding to the optimal G/F was 20.43% (R² = 0.99). For broilers aged 32–42 d, ADG (Fig. 2‑C) showed linear (*P*_linear_ <0.05) and quadratic (*P*_quadratic_ <0.05) responses to increasing SID CP levels. The SID CP level at the plateau breakpoint for ADG was 18.06% (R² = 0.91), and the SID CP level corresponding to the optimal ADG was 19.40% (R² = 0.99).

### Determination of apparent metabolizability of various nutrients

As shown in Table 7, for broilers aged 21–32 d, with the decrease in dietary SID CP levels, the apparent metabolic rate of dry matter exhibited both linear (*P*_linear_ <0.05) and quadratic responses (*P*_quadratic_ <0.01). Meanwhile, increasing dietary SID CP levels resulted in both linear (*P*_linear_ <0.01) and quadratic responses (*P*_quadratic_ <0.01) in the apparent metabolic rate of crude protein. Furthermore, with decreasing SID CP level, the apparent metabolic rate of organic matter demonstrated linear (*P*_linear_ <0.01) and quadratic (*P*_quadratic_ <0.01) responses.

**Table 7:** The impact of SID CP content on nutrient digestibility and serum biochemical indices in broilers.

Items	SID CP levels					SEM^1^	P-value	
	21.00	19.875	18.75	17.625	16.50		Linear	Quadratic
Nutrient digestibility, %								
Dry matter	75.21	75.15	75.12	75.24	75.28	0.02	0.04	<0.01
Crude protein	64.06	66.68	66.09	69.37	71.35	0.59	<0.01	<0.01
Organic matter	76.52	76.40	76.58	76.63	76.90	0.05	<0.01	<0.01
Serum biochemical indices								
Total protein, g/L	26.05	27.63	26.39	26.16	26.83	0.41	0.97	0.98
Albumin, g/L	14.18	14.43	14.10	13.32	13.71	0.18	0.17	0.40
Urea nitrogen, mmol/L	0.42	0.48	0.43	0.42	0.38	0.01	0.08	0.06
Glucose, mmol/L	13.02	12.87	12.60	13.21	13.23	0.13	0.44	0.42
Total cholesterol, mmol/L	3.14	3.15	2.80	2.87	3.06	0.05	0.25	0.11
Triglycerides, mmol/L	0.46	0.43	0.43	0.43	0.44	0.01	0.54	0.67
Non-esterified fatty acids, mmol/L	1.21	1.01	0.86	0.41	0.58	0.07	<0.01	<0.01
Pyruvic acid, µmol/mL	0.46	0.41	0.30	0.27	0.21	0.02	<0.01	<0.01
Total amino acids, µmol/mL	17.22	17.09	16.91	18.26	16.74	0.25	0.91	0.84

1 Standard error of the mean, n = 6.

### Serum biochemical indices

As shown in Table 7, for 32–42 d broilers, the levels of FFA and Pyr exhibited both linear (*P*_linear_ <0.01) and quadratic responses (*P*_quadratic_ <0.01) to dietary SID CP levels. With the increase in dietary SID CP levels, urea concentration showed a quadratic trend (*P*_quadratic_ <0.10). In addition, the serum levels of TP, ALB, GLU, TC, TG, and TAAs did not change significantly (*P* >0.05) with the variation in dietary SID CP levels.

### Slaughter performance and meat quality

The slaughter performance and meat quality of the three treatment groups (HP, MP, and LP) were analyzed. As shown in Table 8, with the decrease in dietary SID CP level, the dressing percentage of broilers aged 32–42 d showed a quadratic response (*P*_quadratic_ <0.05). The data indicated that there were no significant differences (*P* >0.05) in the eviscerated yield percentage during the 32–42 d growth phase. Similarly, no significant differences (*P* >0.05) were observed in drip loss, cooking loss, or shear force of the meat among the various treatment groups.

**Table 8:** The impact of SID CP content on slaughter characteristics and carcass traits and meat quality of 32**–**42 days broilers.

Items	SID CP levels			SEM^1^	P-value	
	21.00	18.75	16.50		Linear	Quadratic
Dressing percentage, %	94.84	96.11	94.86	0.24	0.97	0.02
Eviscerated yield rate, %	75.96	76.23	75.54	0.34	0.66	0.51
Drip loss, %	13.67	14.25	13.30	0.34	0.68	0.31
Cooking loss, %	3.30	3.07	3.39	0.15	0.81	0.42
Shear force	19.31	18.66	19.70	0.32	0.62	0.25

1 Standard error of the mean, n = 6.

### Cecal microbiota

**Fig. 3:**
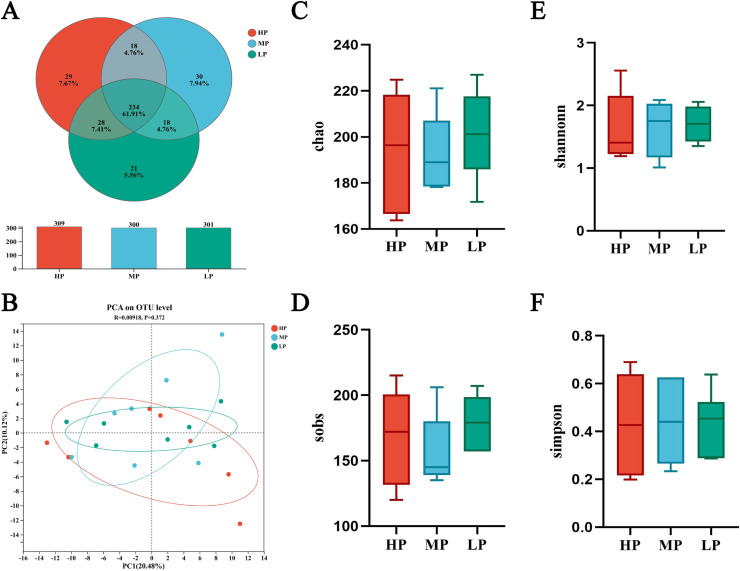
Effects of different SID CP levels on cecal microbial diversity: Venn diagram of species among the three treatment groups (A), PCA of OTUs based on Euclidean distance (B), and Chao (C), Sobs (D), Shannon (E), and Simpson (F) indices. In the figure, HP represents the high SID CP group (SID CP level: 21%); MP represents the medium SID CP group (SID CP level: 18.75%); LP represents the low SID CP group (SID CP level: 16.5%).

Three treatment groups (HP, MP and LP) were selected for analysis of intestinal microbial diversity. As shown in Fig. 3-A, there were 234 shared OTUs among the HP, MP, and LP groups, with 29 OTUs unique to the HP group, 30 OTUs unique to the MP group, and 21 OTUs unique to the LP group. As shown in Fig. 3-B, PCA analysis demonstrated that the microbial community structures were largely similar across the three treatment groups, with no significant variation detected. Alpha diversity analysis (Fig. 3-C and -D) showed that the Sobs and Chao indices decreased quadratically with increasing SID CP levels, with the lowest values observed in the MP group, whereas the Shannon index remained unchanged across groups. The Simpson index showed a linear decrease with increasing SID CP levels, with lower values in the LP group compared with the HP group.

**Fig. 4:**
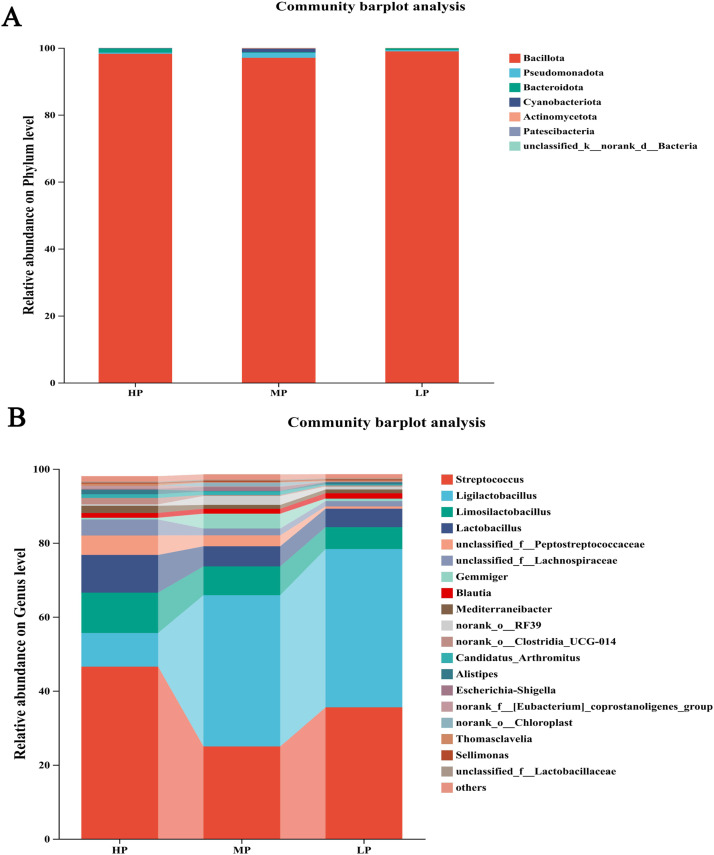
Effects of SID CP Levels on the Microbial Community Composition of Cecum at the Phylum (A) and Genus Levels (B). In the figure, HP represents the high SID CP group (SID CP level: 21%); MP represents the medium SID CP group (SID CP level: 18.75%); LP represents the low SID CP group (SID CP level: 16.5%).

Based on the species annotation results, the top 10 species with the highest relative abundance in each group were selected to generate a bar chart of species relative abundance (Fig. 4-A). The dominant phylum in the broiler intestines was *Bacillota*.

The top 20 species with the highest relative abundance at the genus level were selected for each group to generate a bar chart of species relative abundance (Fig. 4-B). The dominant genera in the broiler intestines were *Streptococcus, Ligilactobacillus, Limosilactobacillus*, and *Lactobacillus*. With increasing SID CP levels, the relative abundances of *Streptococcus* and *Limosilactobacillus* exhibited the lowest values observed in the MP group. The relative abundance of *Lactobacillus* showed a linear decrease as SID CP levels increased, with the highest abundance in the LP group. In contrast, the relative abundance of *Ligilactobacillus* showed a linear increase with increasing SID CP levels, with the highest abundance in the HP group.

**Fig. 5:**
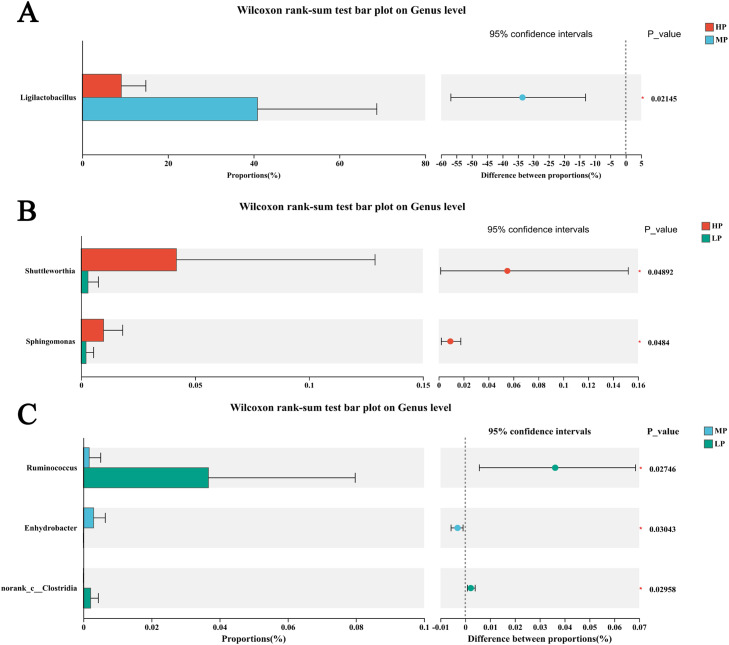
Effects of protein level on species differences in cecal microbiota between the HP and MP groups (A), HP and LP groups (B), and MP and LP groups (C). In the figure, HP represents the high SID CP group (SID CP level: 21%); MP represents the medium SID CP group (SID CP level: 18.75%); LP represents the low SID CP group (SID CP level: 16.5%).

Then, the species differences among the three treatment groups were analyzed (Fig. 5-A,B, and C). With increasing SID CP levels, the relative abundance of *Shuttleworthia* decreased (*P* < 0.05), with the lowest abundance observed in the LP group. The relative abundance of *Ligilactobacillus* peaked in the MP group (*P* < 0.05). The relative abundance of *Ruminococcus* decreased with increasing SID CP levels (*P* < 0.05), with the highest abundance observed in the LP group.

## Discussion

In modern broiler production, the widespread adoption of feed-grade amino acids and increasingly stringent nitrogen emission regulations have driven the formulation of low-CP diets that meet the requirements for SID EAA. However, the CP metric fails to account for variations in protein digestibility among different feed ingredients, while the SID amino acid formulation approach focuses exclusively on individual EAA. Consequently, neither strategy adequately assesses the broiler's overall requirement for total digestible nitrogen. This may lead to an insufficient supply of digestible nitrogen in the diet, thereby constraining the synthesis of endogenous NEAA (Parsons and Rochell, 2025). Therefore, to fill this knowledge gap, SID CP is proposed in this study as a key nutritional parameter. It offers a more accurate assessment of utilizable dietary nitrogen by integrating ingredient-specific SID coefficients. This approach not only fulfills EAA requirements but also secures sufficient nitrogen for endogenous NEAA synthesis, thereby adhering to the fundamental tenet of nitrogen balance within the ideal protein theory. Currently, systematic experimental research on the stage-specific dynamic SID CP requirements for white-feathered broilers remains largely unexplored. Existing recommendations are predominantly derived from literature reviews rather than targeted experimental studies. Therefore, the present study was conducted to investigate the effects of different SID CP levels on growth performance, meat quality, and cecal microbiota of broilers. These findings are expected to provide essential data to help develop feed formulas that are low in crude protein, efficient with nitrogen. On the other hand, they will also create a scientific foundation for the flexible use of a wider range of feed ingredients without compromising good growth performance.

In this study, a commercial enzyme complex (containing cellulase, xylanase, protease, and amylase) was included in all diets at 0.03% to simulate practical production conditions. Enzyme complexes are commonly used in broiler feed to improve nutrient digestibility and growth performance. However, in the context of a requirement study, the addition of exogenous enzymes may potentially confound the estimation of SID CP requirements by enhancing amino acid digestibility. Nevertheless, the consistent inclusion of the enzyme complex across all dietary treatments ensures that the relative comparisons among SID CP levels remain valid. Therefore, the results of this study are applicable to commercial settings where this enzyme complex is used as standard practice.

This study revealed that maintaining an appropriate dietary SID CP level is crucial for optimizing the growth performance of broilers. During the 0–21 d period, broiler growth performance was observed to be positively correlated with the experimental SID CP levels. Notably, within the range of 10.5% to 21.5% SID CP, growth performance consistently improved as SID CP increased. This indicates that, within the range tested, the growth potential of the broilers had not reached a plateau, and SID CP was the primary nutritional factor limiting their performance. This finding differs from the classical nutrient requirement theory based on traditional corn-soybean meal-based diets. A study suggested that the dietary crude protein level for male broilers aged 0–10 d should be 22% (Yu et al., 2024). In conventional diets, where ingredient protein digestibility is typically high and consistent, a strong correlation exists between CP and SID CP. Consequently, CP often serves as a reliable proxy indicator for SID CP under such conditions. Traditional low-CP diets are typically formulated by reducing soybean meal and increasing corn to lower the CP content. However, with further research, an increasing number of studies on low-CP diets have begun to explore the use of unconventional feed ingredients to partially replace corn and soybean meal, aiming to further reduce feed costs and improve resource utilization efficiency. The protein digestibility of these ingredients exhibits substantial variation, ranging from 30%–95% (National Research Council, 1994; Applegate and Angel, 2014). Furthermore, the presence of anti-nutritional factors can significantly impair protein digestion and absorption. This study demonstrated that a low-protein diet formulated with non-conventional ingredients at a CP level of 21.5%, yet possessing low digestibility, could yield a considerably lower actual SID CP than a conventional diet with a CP of 20% but high digestibility. The observed linear relationship between growth performance and SID CP confirms that, within complex feed formulation systems, SID CP is a more precise nutritional metric than crude protein. Broilers aged 0–21 d are in a phase of rapid growth, characterized by significant organ development and substantial potential for protein deposition. This results in an exceptionally high demand for utilizable nitrogen sources, compounded by their immature digestive system. Therefore, providing a higher SID CP level during this stage can more effectively meet the broilers' requirements for digestible amino acids. This, in turn, directly stimulates in vivo protein synthesis, leading to improved daily gain and feed conversion efficiency (Martinez et al., 2025).

For the period 21–42 d, there was a significant quadratic relationship between the broilers' ADG and the level of SID CP in their diet. Based on model fitting, optimal growth performance was achieved in broilers at SID CP levels of 19.00% and 19.40% during the 21–32 and 32–42 d of age periods, respectively. The period from 21 to 42 d of age in broilers is characterized by a maturing digestive system (Ranjitkar et al., 2016), a relative deceleration in growth rate, and consequently, a metabolism that is more sensitive to the efficiency and balance of nutrient utilization. In this experiment, the dietary SID CP level showed a distinct optimal range, which varied across growth stages. For broilers aged 21–32 d, a SID CP range of 19.00%–20.43% resulted in better growth performance, while for broilers aged 32–42 d, the corresponding range was 18.06%–19.40%. When SID CP fell below this optimum, growth was constrained by insufficient substrates for protein synthesis, a pattern similar to that observed during the 10–21 d of age period. Conversely, when SID CP exceeded the optimal level, growth performance declined, which is generally associated with imbalances in amino acid metabolism or an inappropriate energy-to-protein ratio (Gous et al., 2018; Cordero et al., 2025). When dietary protein levels are excessively high, a greater proportion of energy metabolism substrates is derived from amino acid oxidation, although carbohydrates remain the primary energy source (Luczkowska et al., 2024). Simultaneously, the catabolism of the surplus protein significantly increases the nitrogen excretion load (Oliva et al., 2019), thereby elevating the metabolic burden on the liver and kidneys, while also possibly diverting physiological resources that could otherwise be utilized for other energy metabolic pathways or fat deposition. The observed quadratic response of G/F to SID CP levels underscores the organism's increased reliance on a precisely balanced protein supply during this period.

From the perspective of nutrient metabolism, a moderate reduction in dietary SID CP level, while maintaining amino acid balance, instead increased the apparent metabolic rates of crude protein, dry matter and organic matter. In this experiment, gradually lowering the SID CP level while supplementing synthetic essential amino acids brought the dietary amino acid pattern closer to the ideal requirements of broilers at this growth stage. This reduced catabolism caused by amino acid imbalance or unnecessary excess, thereby directing more of the ingested protein toward protein synthesis, which was reflected in a systematic improvement in the apparent metabolic rate of crude protein—consistent with previous studies (Ma et al., 2022; Li et al., 2025). Furthermore, similar to the previous research, the apparent metabolic rate of dry matter in this trial showed a slight linear and quadratic increase as the SID CP level decreased (Heo et al., 2023). The dry matter metabolic rate primarily reflects the utilization of carbohydrates and fats. Excessively high SID CP levels may increase the metabolic burden on the body, thereby slightly inhibiting the digestion of dry matter and the efficiency of energy metabolism (Peres et al., 2022). In addition to the increased metabolic burden, excessively high SID CP levels can also negatively affect gut health. When dietary protein supply exceeds the bird's requirements for protein synthesis, the surplus undigested protein enters the hindgut and undergoes fermentation by the cecal microbiota. This proteolytic fermentation process generates potentially toxic metabolites, including ammonia, biogenic amines, indole, and phenolic compounds (Bryan et al., 2020; Sung and Adeola, 2025). Thus, the negative impact of excessive SID CP levels on broiler performance may be partially attributed to both the systemic metabolic burden and the local production of harmful cecal protein. When the SID CP level was reduced to an appropriate range, the overall metabolic stress was alleviated, allowing the body to utilize the non-protein energy components of the diet more fully.

Serum biochemical indicators reflect the body's metabolic status and nitrogen utilization efficiency. When the dietary SID CP level was around 19.4% during the 32–42 d period, the urea nitrogen results indicated that the ingested amino acids were efficiently utilized for protein deposition, with relatively less ammonia produced from deamination. Consequently, the urea nitrogen level had not yet peaked. When the dietary SID CP approached or exceeded this level, reaching 19.875%, protein synthesis reached saturation. The excess amino acids were forced into oxidation for energy, leading to a sharp increase in deamination. At this point, urea nitrogen synthesis peaked, signifying a significant increase in nitrogen waste and diminishing marginal returns of dietary SID CP. The results for free fatty acids and pyruvate suggested that the energy metabolism pattern of broilers shifted as the dietary SID CP level increased. With higher dietary SID CP levels, the breakdown and conversion processes of excess amino acids consumed substantial energy and potentially interfered with the normal energy metabolic pathways of glucose and fats (Mansoori et al., 2025). To meet these additional energy demands, the body was compelled to mobilize stored fat, resulting in a continuous rise in serum free fatty acid levels. Meanwhile, the level of Pyr showed a linear increase. Pyr is a key intermediate that helps convert glucogenic amino acids into glucose and sits at the crossroads of glycolysis, gluconeogenesis, and amino acid metabolism (Holeček, 2024). This indicated that as the SID CP level increased, the rate of pyruvate generation from amino acid conversion exceeded the rate of its utilization in subsequent metabolic pathways, leading to an accumulation of this intermediate. The results demonstrate that excessively high SID CP levels reduce overall energy metabolism efficiency and increase metabolic burden, whereas at the optimal SID CP level identified in this trial, broilers were able to achieve optimal growth performance within their metabolic thresholds.

Carcass traits such as dressing percentage and eviscerated yield provide a direct assessment of broiler production performance (Huang et al., 2016). Meanwhile, drip loss, cooking loss, and shear force reflect meat quality. The study found that within the tested SID CP range, moderately reducing the SID CP level resulted in the optimal dressing percentage without negatively affecting key meat quality indicators. This indicates that through precise amino acid nutrition regulation, it is possible to optimize carcass composition and reduce feed protein costs while ensuring the final product's eating quality and processing characteristics.

The cecum hosts the highest microbial abundance and diversity within the avian intestinal tract, and its microbiota plays a crucial role in animal growth performance, immune response, and overall health (Bindari and Gerber, 2022). In this experiment, three treatment groups with different dietary SID CP levels—HP (21% SID CP), MP (18.75% SID CP), and LP (16.5% SID CP)—were analyzed. The results showed that the overall structure of the microbial community was not significantly changed by the different SID CP levels.

Alpha diversity indices revealed that with increasing SID CP levels, the Sobs and Chao indices exhibited a quadratic response, with the lowest values observed in the MP group. This indicates a reduction in species richness while the evenness among the remaining species was maintained. In contrast, the Shannon index remained unchanged across groups. The Simpson index showed a linear decrease with increasing SID CP levels, with lower values in the LP group compared with the HP group. The Simpson index is highly sensitive to shifts in the abundance of the most dominant taxa within a community (Nordberg et al., 2009). These results suggest that when the protein level was lowered to 16.5%, although the overall taxonomic composition was preserved, resource availability may have approached the lower threshold for certain key dominant microbial groups, leading to a decline in their relative abundance.

To further investigate the microbial compositional characteristics underlying the aforementioned changes, we conducted an analysis at the phylum level. At this level, the dominant phylum in the broiler intestine was *Bacillota*, one of the primary fermenters of dietary fiber within the gut. The fermentation process produces short-chain fatty acids (**SCFAs**), such as butyrate, acetate, and propionate. Butyrate serves as the main energy source for intestinal epithelial cells, thereby promoting gut health (Facchin, 2024). In contrast, acetate and propionate are absorbed and are directly involved in the host's energy metabolism and lipid synthesis (He et al., 2020). Given that broiler diets are rich in starch and non-starch polysaccharides, and the intestinal tract is relatively short with a rapid digesta passage rate, all three treatment groups exhibited a high relative abundance of *Bacillota*. This confirms the robust ecological niche of *Bacillota* as a critical gateway for energy acquisition in the broiler intestine (Diaz Carrasco et al., 2019; Deryabin et al., 2024).

Although the overall microbial community structure at the phylum level remained stable, the effect of different protein levels on the microbial community was more clearly seen in changes at the genus level. At the genus level, the richer protein substrates and balanced nutrition in the MP diet likely promoted the proliferation of facultatively metabolic commensals represented by *Ligilactobacillus*. While utilizing amino acids, these bacteria may also contribute to the maintenance of intestinal health. The increase in *Ligilactobacillus* could be associated with a more stable gut environment and enhanced carbohydrate fermentation (Yang et al., 2023; Bedir et al., 2025). It should be noted that the reduction in dietary SID CP levels was accompanied by an increase in dietary fiber content due to the greater inclusion of corn and reduced soybean meal. As the SID CP level decreased, the LP diet contained higher levels of structural carbohydrates, increasing the host's demand for efficient digestion of non-protein energy. This typically led to an enrichment of obligate fiber-degrading bacteria in the gut microbiota, represented by *Ruminococcus* (Wang et al., 2018; Sharma et al., 2025). Simultaneously, changes in the form and total amount of dietary nitrogen directly altered physicochemical conditions in the intestine, such as pH and substrate composition, ultimately shaping distinct microbial habitats. This allowed bacteria like *Ruminococcus*, which could adapt to the new environment, to gain a growth advantage.

These results indicate that structural shifts in the gut microbial community are a key strategy for functionally adapting to dietary nutritional composition. The balanced symbiotic pattern observed in the MP group suggests advantages in maintaining intestinal homeostasis and supporting multi-dimensional nutrient metabolism. The reduction in SID CP levels induces a structural shift toward a specialized fiber-degradation mode, which represents a metabolic compensation under protein limitation, aimed at maximizing the energy value of dietary fiber. Therefore, from the perspective of microbial ecological function, the microbiota structure shaped by the MP diet exhibits both metabolic comprehensiveness and systemic stability. This protein level not only meets the direct nutritional needs of the host but also supports an intestinal microecosystem that efficiently promotes host health and metabolic balance.

## Conclusion

It was hypothesized that an optimized reduction in SID CP content could improve growth and nutrient utilization without compromising carcass traits or meat quality, while potentially modulating the abundance of specific bacterial taxa in the cecum. This experiment was conducted to investigate the effects of different SID CP levels on the growth performance, meat quality, and cecal microbiota of Arbor Acres broilers by feeding diets with graded SID CP levels. In this experiment, the level of SID CP in the diet significantly influenced the growth performance of AA broilers from d 0–42. From d 0–21, there was a clear positive correlation between SID CP levels and growth performance. As SID CP increased, both growth performance and feed efficiency improved. During the period from 32 to 42 d of age, growth performance improved with increasing SID CP, but plateaued after the SID CP level approached approximately 19.4%, which was the estimated breakpoint from the model. Serum FFA and Pyr concentrations increased with increasing SID CP levels, while other plasma biochemical parameters were not significantly affected. SID CP levels did not significantly influence carcass characteristics, meat quality, or gut microbial diversity. However, it influenced the relative abundance of specific bacterial genera. Therefore, in practical feed formulation, the SID CP level can be precisely adjusted according to the growth stage. In conclusion, these findings suggest that optimizing SID CP supply can maintain growth performance while reducing the metabolic burden associated with excess nitrogen.

## CRediT authorship contribution statement

**Shurui Chen:** Writing – original draft, Visualization, Validation, Methodology, Investigation, Data curation. **Ruiping Liang:** Writing – review & editing, Formal analysis. **Min Fan:** Supervision. **Lin Lu:** Supervision. **Junyan Zhou:** Writing – review & editing, Supervision, Resources, Project administration, Funding acquisition.

## Disclosures

The authors declare that they have no known competing financial interests or personal relationships that could have appeared to influence the work reported in this paper.
